# Association between Interleukin-17F 7488A/G and 7383A/G polymorphisms and susceptibility to juvenile idiopathic arthritis

**DOI:** 10.1038/s41390-022-02288-1

**Published:** 2022-09-06

**Authors:** Eman Rateb Abd Almonaem, Ashraf Mohamed Shaheen, Amira M. N. Abdelrahman, Waleed A. Hassan, Noha Mohamed Daay El Khair, Omima Mohamed Abdel Haie

**Affiliations:** 1 Pediatric Department, Faculty of Medicine, Benha University, Benha, Egypt; 2 Clinical and Chemical Pathology Department, Faculty of Medicine, Benha University, Benha, Egypt; 3 Rheumatology, Rehabilitation, and Physical Medicine Department, Benha University, Benha, Egypt; 4 M.B.B.Ch. Faculty of Medicine, Benha University, Benha, Egypt

## Abstract

**Background:**

Interleukin-17F (IL-17F), one of the cytokines, is crucial in the pathophysiology of juvenile idiopathic arthritis (JIA). Therefore, we aimed to determine the relation between IL17F 7488A/G and IL17F 7383A/G single-nucleotide polymorphisms and JIA susceptibility and to explain their impact on the disease activity.

**Methods:**

Genomic DNA of 70 patients with JIA and 70 age and sex-matched controls were extracted and typed for IL17F 7488A/G and IL17F 7383A/G single-nucleotide polymorphisms, using polymerase chain reaction with sequence-specific primers method, and compared between patients and controls.

**Results:**

When compared to AA participants, children with the AG genotype of the IL17F 7488A/G and IL17F 7383A/G polymorphisms showed a substantially greater risk of JIA. Furthermore, children with the G allele were 2.8 folds more likely to have JIA than the A allele for IL17F 7488A/G polymorphism and 3.72 folds for IL17F 7383A/G polymorphism. Children with AG genotype of IL17F 7383A/G polymorphism were far more likely to have high activity JIA.

**Conclusions:**

The G allele of both IL17F 7488A/G and IL17F7383 A/G polymorphisms is associated with increased JIA susceptibility, and JIA at High Disease Activity was more likely to develop in AG subjects of the IL17F 7383 A/G polymorphism.

**Impact:**

- The relationship between Interleukin-17F 7488A/G and 7383A/G polymorphisms and risk for JIA has not been recognized before.
- Impact of Interleukin-17F 7488A/G and 7383A/G genotypes on JIA disease activity.
- The G allele of both IL17F 7488A/G and IL17F7383 A/G polymorphisms are associated with increased JIA susceptibility.
- AG genotype of Interleukin-17F 7383 A/G polymorphism compared to AA patients, had a higher probability of developing JIA at a High Disease Activity (HDA) level.

## Introduction

Juvenile idiopathic arthritis (JIA) points to a string of unexplained etiology of inflammatory arthritis that occurs earlier than 16 years of age and with 6 weeks duration as a minimum.^1^ JIA is divided into 7 subtypes by the International League of Associations for Rheumatology classification system (second revision): systemic arthritis, oligoarthritis, rheumatoid factor (RF)-positive polyarthritis, RF-negative polyarthritis, enthesitis-related arthritis, psoriatic arthritis, and undifferentiated arthritis.^2^ The precise etiology of JIA is still not completely clear; however, a better understanding of the disease was achieved due to the latest advances in molecular biology in the last decade.^3^

Cytokines engage and play essential roles in the development of JIA. Such cytokines are IL-10, IL-1b, IL-6, IL-17, and tumor necrosis factor-alpha (TNFa).^4–8^ While there are six identified isoforms of IL-17, from A to F, Th17 cells can only produce IL-17A and IL-17F,^9^ and they are both pro-inflammatory cytokines. Recent research has demonstrated that IL-17A and/or IL-17F play a role in the development of inflammation in a variety of disorders, particularly in autoimmune conditions like rheumatoid arthritis (RA), psoriasis, juvenile idiopathic arthritis (JIA), Crohn’s disease and various others.^9–12^ It has been suggested that the previously mentioned immune mediators with polymorphic gene sequences represent potential markers of an individual’s susceptibility to JIA.^13–15^

Up to now, in the context of rheumatologic disorders, such as juvenile-onset systemic lupus erythematosus, many single-nucleotide polymorphisms (SNPs) in various cytokine genes, impacting their level of synthesis, have been researched.^16–19^ Nevertheless, as far as we know, there has been no association study conducted on interleukin-17 gene cluster and interleukin-17 receptor polymorphisms in an Egyptian population with JIA. The current study aimed to investigate IL17F as a potential candidate gene for JIA and to assess the relationship between IL17F polymorphisms 7488A/G and 7383A/G and JIA risk in the Egyptian population. Additionally, we sought to elucidate their impact on the activity of the disease.

## Subjects and methods

### Study design and population

In the current study, we enrolled a total of 70 JIA patients recruited from the Rheumatology Clinic of Rheumatology and Pediatrics Department, Benha University, between September 2019 and November 2021. These patients were considered the case group and were compared to 70 healthy unrelated sex and age-matched controls. We used the ILAR classification criteria for JIA to demonstrate the diagnosis of JIA.^2^ Patients with recent infection, malignancy, other autoimmune diseases, DM, or IBD were excluded. Before enrollment in the study, parents/guardians of both JIA patients and controls delivered written consent. The study protocol was approved by The Ethical Scientific Committee of the Faculty of Medicine, Benha University, conferring to the World Medical Association Declaration of Helsinki.^20^

A detailed history was obtained from all JIA patients with a thorough clinical examination while focusing specifically on the pattern and distribution of joint involvement, uveitis, and other extra-articular findings and medications. The performance of Juvenile arthritis disease activity score (JADAS27)^21^ is uncertain in systemic-onset arthritis,^22^ hence it was assessed in patients with oligoarticular and polyarticular involvement.

As per Beukelman et al.^23^, we graded the activity of the disease into high, moderate, and low, while the inactive disease was recognized using Wallace criteria.^24^ We used the Juvenile Arthritis Multidimensional Assessment Report (JAMAR)^25^ to assess the functional condition and quality of life (QoL) in JIA patients. Either the patient or their parent may report the compound scale composed of 15 items. The assessment involved a physical function (PF) scale (0–45), pain visual analog scale (VAS) (0– 10), physician assessment of disease activity (0–10), health-related (HRQoL) (0–30) and self-assessment of patient’s overall well-being (0–10). We used the Systemic Juvenile Arthritis Disease Activity Score (sJADAS)^26^ and Modified Systemic Manifestation Score to measure disease activity levels in systemic-onset JIA (sJIA).^27^

### Sampling

Under complete aseptic conditions, five milliliters of venous blood were collected from all subjects. Then, in tubes containing EDTA anticoagulants, 2 ml of the blood were added and consequently divided into two tubes:

- A tube for complete blood count was conducted by an automated hematology analyzer (Sysmex XS -500 i, Japan).
- The other was stored at –80 °C until used for DNA extraction and IL17F 7383 G allele and 7488 G allele genotyping.

The remaining blood was emptied into plain tubes for serum preparation and was stored at −80 °C to be used for assessment of:

- C-reactive protein (CRP) by CRP-Latex Slide Agglutination supplied by (SPINREACT Spain).
- Erythrocyte sedimentation rate (ESR) using the Westergen method.
- Rheumatoid factor (RF) by nephelometry using MISPA-i2 kit supplied by AGAPPE Diagnostics, Kerala, India.
- Serum ferritin by enzyme-linked immunosorbent assay (ELISA; R&D Systems, Minneapolis, MN).

### Genomic DNA extraction and genotyping

DNA purification kit (Invitrogen; Thermo Fisher Scientific, Inc) was used for genomic DNA isolation from peripheral blood leukocytes as per manufacturer instructions.

By RT_PCR, the human IL17F gene was amplified using the primer set (Biosearch technologies) that comprised a 5’GTGTAGGAACTIGGGCTGCATCAAT 3’) (forward primer) and 5’AGCTGGGAATGCAAACAAAC 3’ (reverse primer) for IL17F 7383 A > G, SNP rs2397084 and 5’ GTGTAGGAACTTGGGCTGCATCAAT 3’) (forward primer) and 5’ AGCTGGGAATGCAAACAAAC 3(reverse primer) for IL17F 7488 A > G, SNP rs763780 ‘generating an amplicon of 470 length. By using PCR thermal cycler (Piko-Real 24 Thermo Fisher Scientific, Finland), PCR was generated.

**Fig. 1:**
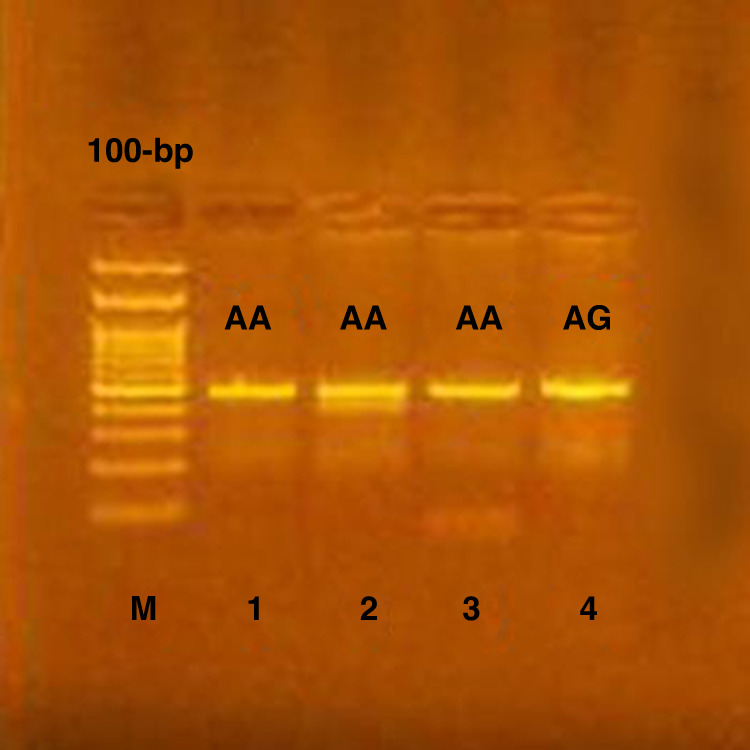
Agarose gel electrophoresis of IL-17F 7383 polymorphism. Genotyping of the gene IL-17F 7383 gene polymorphism, lane (1,2,3) showed the homozygous AA genotype, while lane (4) showed heterozygous AG genotype.
- Two bands were produced at 418 and 52 bp with DNA from individuals with IL17F7488 homozygous GG genotype for IL-17F rs763780 polymorphism. While three bands were produced by an individual with Homozygous AA genotype at (288,130 and 52 bp). Furthermore, four bands were produced at (418,52,288 and130 bp) with Heterozygous AG genotype (Fig. 2).
  - In each experiment, Internal positive and negative controls were included. To confirm validation, 10% of the samples were selected and replicated randomly. Furthermore, two independent investigators read the gel.

- Following the preliminary denaturation step at 94 °C for 8 min, the mixture was exposed to 35 cycles of denaturation at 94 °C for 50 s, annealing for 50 s at 59 °C, and 72 °C for 1 min followed by a final extension at 72 °C for 5 min. Following PCR, digestion of the reaction mixture was conducted with AvaII restriction endonuclease) (New England BioLabs Inc.) and NlaIII restriction endonuclease (New England BioLabs Inc.) for IL17F7383 and IL17F 7488, respectively. To resolve the digest mixture, 2% agarose gel stained with ethidium bromide was used. For IL-17F7383A > G rs 2397084 polymorphism, the DNA from individuals with IL17F7383 homozygous GG genotype produced two bands at 395 and 75 bp, while Homozygous AA genotype produced one single band at 470 bp while, and Heterozygous AG genotype produced three bands at 470,395 and 75 bp (Fig. 1).

**Fig. 2:**
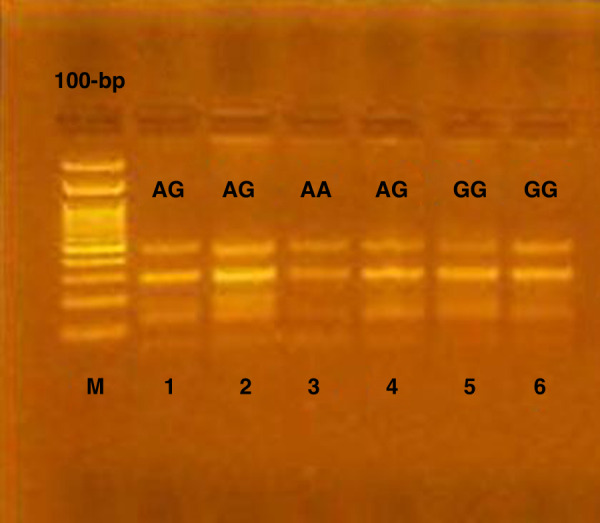
Agarose gel electrophoresis of IL-17F 7488 polymorphism. Genotyping of the gene IL-17F 7488 polymorphism M.PCR marker (DNA ladder 100 bp), lane (5,6) showed homozygous GG genotype, lane (3) showed homozygous AA genotype while lane (1,2,4) showed heterozygous AG genotype.

### Statistical analysis

The data were analyzed using SPSS software, version 22.0 (IBM, Armonk, NY), and MedCalc software (MedCalc Software, Mariakerke, Belgium) for Windows. Categorical data were presented as numbers and percentages, and Chi-square (*χ*^2^), and Fisher’s exact tests were used to analyze them. The odds ratio (OR) and the corresponding 95% confidence intervals (95% CI) were calculated. Using the Shapiro–Wilks test, quantitative data were tested for normality assuming normality at *p* > 0.05. Normally distributed variables were expressed as mean ± standard deviation and analyzed by the Student “*t*” test for two independent groups, while nonparametric ones were presented as median and inter-quartile range (IQR), and analyzed by Mann–Whitney *U*-test. *P* ≤ 0.05 was considered significant. Genotype distributions in the studied groups were in Hardy–Weinberg equilibrium for gene polymorphisms (data not shown). Hardy–Weinberg equilibrium was calculated according to OEGE—Online Encyclopedia for Genetic Epidemiology studies.^28^

## Results

The mean age of the 70 patients was 7.3 ± 2.9 years; 47 females and 23 males (F: M 2.04:1); BMI was 15.8 ± 0.81. The mean age (7.1 ± 3.1 years), sex (41 females and 29 males; 1.4:1), and BMI (15.9 ± 0.77) of the control were comparable (*p* = 0.7, *p* = 0.2, and *p* = 0.6, respectively) (Table 1).

**Table 1:** Basic characters of the studied groups.

Variable		JIA patients (*n* = 70)	Controls (*n* = 70)	Test of significance	*p*
Age (years)	Mean ± SD	7.3 ± 2.9	7.1 ± 3.1	St.“*t*” = 0.38	0.70 (NS)
	Range	3–12	3–12		
Sex	Female *N* (%)	47 (67.1)	41 (58.6)	*X*^2 ^= 1.1	0.29 (NS)
	Male *N* (%)	23 (32.9)	29 (41.4)		
Weight (kg)	Mean ± SD	24.1 ± 7.9	23.4 ± 8.6	St.“*t*” = 0.49	0.62 (NS)
	Range	14-38	13-38		
Height (cm)	Mean ± SD	121.4 ± 17.8	119.1 ± 20.4	St.“*t*” = 0.71	0.48 (NS)
	Range	93-148	90-148		
BMI (kg/m^2^)	Mean ± SD	15.8 ± 0.81	15.9 ± 0.77	St.“*t*” = 0.45	0.65 (NS)
	Range	14.6–17.3	14.5-17.5		

*JIA* Juvenile idiopathic arthritis, St.“*t*”: student *t*-test, *X*^2^: Chi-square test, BMI body mass index, NS non-significant.

The median duration of JIA disease was 9 months (IQR 1–44 months). 37 (52.9%) were oligoarticular, 21 (30%) polyarticular, and 12 (17.1%) were systemic-onset JIA. None of the patients had psoriatic or enthesitis-related arthritis.

As for disease activity, 30% of patients had an inactive disease, 12.9% had mild disease activity, 28.6% had moderate disease activity, and 28.6% had severe disease activity. 7.1% of patients had a positive rheumatoid factor, while 92.9% had a negative rheumatoid factor. Moreover, 8.6% of cases had uveitis, while 91.4% of cases did not have uveitis. A statistically significant difference between JIA subtypes was present regarding disease duration, disease activity, and rheumatoid factor (*p* = 0.01, *p* = 0.02, and *p* = 0.004, respectively).

The median ESR of JIA patients was 28 mm/h (IQR = 17.4–38.5) and showed statistically significant differences among JIA subtypes (*p* > 0.001), other laboratory parameters did not show any significant differences among JIA subtypes.

Statistically, a significant difference was observed in genotypic distributions of IL17F7488 A/G polymorphism among subtypes of JIA (FET = 13.2 and *p* = 0.004); polyarticular subjects (71.4% AA genotype and 28.6% AG genotype), oligoarticular subjects (64.9% AA genotype and 35.1% AG genotype), and systemic-onset subjects (75% AA genotype and 25% GG genotype). We found no significant difference in the distribution of genotypes among subtypes of JIA for IL17F 7383A/G polymorphisms (*p* > 0.05).

Regarding Juvenile arthritis disease activity score (JADAS27), a significant difference was found between polyarticular and oligoarticular JIA patients (*p* > 0.001). Furthermore, for Juvenile Arthritis Multidimensional Assessment Report (JAMAR) a statistically significant difference was revealed in our results among JIA subtypes in physical function (PF) scale, pain visual analog scale (VAS), physician assessment of disease activity, health-related (HRQoL), and self-assessment of patients’ overall well-being (*p* = 0.009, *p* = 0.02, *p* > 0.001, *p* = 0.003, and *p* = 0.01 respectively).

In addition, our results revealed statistically significant differences regarding Juvenile Arthritis Multidimensional Assessment Report (JAMAR) among JIA subtypes in physical function (PF) scale, pain visual analog scale (VAS), physician assessment of disease activity, health-related (HRQoL), and self-assessment of patients’ overall well-being (*p* = 0.009, *p* = 0.02, *p* > 0.001, *p* = 0.003, and *p* = 0.01, respectively). In systemic-onset JIA patients, the median (IQR) of Modified Systemic Manifestation Score (MSMS) and sJADAs27 were 5 (1–6.8) and 11 (6.5–16.5) (Table 2).

**Table 2:** Characteristics of juvenile idiopathic arthritis (JIA) patients.

Parameter			Juvenile idiopathic arthritis patients (JIA)				
			Total (*n* = 70)	Polyarticular (*n* = 21)	Oligoarticular (*n* = 37)	Systemic (*n* = 12)	*p*
Age (years)			7.3 ± 2.9	7.09 ± 3.11	7.43 ± 3.00	7.25 ± 2.95	**0.92 (NS)**
Sex	F:M		47:23	15:6	26 :1	6:6	**0.49 (NS)**
BMI (kg/m^2^)			15.8 ± 0.8	15.8 ± 0.81	15.8 ± 0.84	15.8 ± 0.79	0.98 (NS)
Disease duration (mo)			9.0 (6–12)	12 (6–17)	7 (4–12)	9 (6–12)	**0.013 (S)**
Activity	Inactive		21 (30)	2 (9.5)	14 (37.8)	5 (41.7)	**0.028 (S)**
	Low activity		9 (12.9)	4 (19)	5 (13.5)	0.0 (0.0)	
	Moderate activity		20 (28.6)	4 (19)	12 (32.4)	4 (33.3)	
	High activity		20 (28.6)	11 (52.4)	6(16.2)	3(25)	
RF	Negative		65 (92.9)	16 (76.2)	37 (100)	12 (100)	**0.004 (S)**
	Positive		5 (7.1)	5 (23.8)	0.0 (0.0)	0 (0.0)	
Uveitis	Negative		64 (91.4)	21 (100)	31 (83.8)	12 (100)	0.063 (NS)
	Positive		6 (8.6)	0 (0.0)	6 (16.2)	0 (0.0)	
ESR (mm/1sth)			28 (17.4–38.5)	23.7 (17.8–30.9)	31.6 (20.6-35.4)	31.5 (44.6–4.4)	**>0.001 (HS)**
CRP (mg/L)			14.1 (7.8–20.7)	14.6 (8.2–20.5)	11.6(7.5-20.3)	33.5 (22.5–53.5)	0.78 (NS)
Hb (gm/dL)			11.8 ± 1.68	11.63 ± 1.23	11.92 ± 1.95	10.93 ± 1.57	0.81 (NS)
WBCs (x10^9^/L)			7.6 ± 1.8	7.69 ± 1.95	7.72 ± 1.66	7.06 ± 2.17	0.54 (NS)
PLTs (x10^9^/L)			229.8 ± 39.2	239.2 ± 49.06	222.0 ± 35.00	237.5 ± 28.4	0.21 (NS)
Ferritin (ng/mL)			391.7 ± 115.6	344.7 ± 55.5	406.0 ± 121.4	429.5 ± 153.5	0.068 (NS)
IL-17F 7488 (A > G)	AA		48 (68.7)	15 (71.4)	24 (64.9)	9 (75)	**0.004 (S)**
	AG		19 (27.1)	6 (28.6)	13 (35.1)	0 (0.0)	
	GG		3 (4.3)	0 (0.0)	0 (0.0)	3 (25)	
IL-17F 7383 (A > G)	AA		50 (71.4)	16 (76.2)	28 (75.7)	6 (50)	**0.21 (NS)**
	AG		20 (28.6)	5 (23.8)	9 (24.3)	6 (50)	
JADAs 27 (0–57)			9 (4–13.7)	15.2 (8–20.5)	7 (3–9.8)	-	**>0.001 (HS)**
JAMAR							
PF score (0–45)			8 (7–10)	20 (7–30)	8 (7–9)	7.5 (6.25–8.75)	**0.009 (S)**
Pain VAS (0–10)			6 (3–8)	6 (6–8)	4 (2–8)	6 (5.3–8)	**0.027 (S)**
Ph activity (0–10)			2 (1–3.5)	3.5 (2–5)	1 (0–2)	3 (1.25–4)	**>0.001 (HS)**
HRQoL (0–30)			3 (2–5)	5 (2.5–7)	2 (1–5)	4.5 (2.25–6.75)	**0.003 (S)**
PW VAS (0–10)			3 (2–4)	3 (1–3)	3 (2–4)	4 (2–4.75)	**0.019 (S)**
MSMS (systemic)^a^			5 (1–6.8)	-	-	5 (1–6.8)	**-**
sJADAs27 (0–67)(systemic)^a^			11 (6.5–16.5)	-	-	11 (6.5–16.5)	**-**

*BMI* body mass index, *RF* rheumatoid factor, *ESR* erythrocyte sedimentation rate, *CRP* C-reactive protein, *Hb* hemoglobin, *WBC* white blood cells, *PlTS* platelets, *IL* interleukin, *JADAS* juvenile disease activity score, *JAMAR* Juvenile Arthritis Multidimensional Assessment Report, *PF* physical function, *VAS* visual analog scale, *Ph* physicians assessment of activity, *HRQoL* health-related quality of life, *PW* patients well-being, *MSMS* Modified Systemic Manifestation Score, *sJADAs* systemic Juvenile Arthritis Disease Activity Score.

Bold values are significant at *p* ≤ 0.05.

^a^reference category.

Regarding IL17F 7488A/G polymorphism, individuals with AG genotype had a significantly higher risk of JIA than AA subjects (OR 3.02; 95% CI, 1.2–7.5; *p* = 0.017). In addition, individuals with the G allele were 2.8 folds more likely to have JIA than the A allele (OR 2.8; 95% CI, 1.3–6.1; *p* = 0.008). Similarly, with IL17F7383 A/G polymorphism, individuals with AG genotype had a significantly higher risk of JIA than AA subjects (OR 4.27; 95% CI,1.59–11.4; *p* = 0.002). Furthermore, individuals with the G allele were 3.72 folds more likely to have JIA than the A allele (OR 3.72; 95% CI, 1.4–9.5; *p* = 0.006) (Table 3).

**Table 3:** Comparing the studied groups regarding IL-17F 7488 gene polymorphism.

IL7SNPs	Genotypes	Allele	Cases *N* (%)	Controls *N* (%)	OR (95%CI)	*p*
IL-17F 7488 (A > G)	AA		48 (68.7)	61 (87.1)	Ref	----
	AG		19 (27.1)	8 (11.4)	3.02 (1.2–7.5)	=0.017 (S)
	GG		3 (4.3)	1 (1.4)	3.8 (0.4–37.8)	0.25 (NS)
		A	115 (82.1)	130 (92.9)	2.8 (1.3–6.1)	0.008 (S)
		G	25 (17.9)	10 (7.1)		
IL-17F 7383 (A > G)	AA^a^		50 (71.4)	64 (91.4)	4.27 (1.59–11.4)	0.002 (S)
	AG		20 (28.6)	6 (8.6)		
	GG		0 (0.0)	0 (0.0)		
		A^a^	120 (85.7)	134 (95.7)	3.72 (1.4–9.5)	0.006 (S)
		G	20 (14.3)	6 (4.3)		

*IL* interleukin, *S* significant, *NS* non-significant, *OR* odds ratio.

^a^reference category.

A significantly higher risk to develop high activity JIA compared to AA subjects (*p* = 0.009) was observed in individuals with AG genotype of Interleukin-17F 7383A/G polymorphism, and no significant association was observed between genotype distributions of Interleukin-17F 7488A/G polymorphism and JIA activity (*p* = 0.95) (Table 4).

**Table 4:** Disease activity according to IL17F 7383 gene polymorphism.

		IL17F 7383 (A > G)		IL17F 7488 (A > G)		
		AA	AG	AA	AG	GG
Total *N* (%)		50 (100.0%)	20 (100.0%)	48 (100.0%)	19 (100.0%)	3 (100.0%)
Activity	Inactive *N* (%)	14 (28.0%)	7 (35.0%)	16 (33.3%)	4 (21.1%)	1 (33.3%)
	Low activity *N* (%)	7 (14.0%)	2 (10.0%)	6 (12.5%)	3 (15.8%)	0 (0.0%)
	Moderate activity *N* (%)	19 (38.0%)	1 (5.0%)	12 (25.0%)	7 (36.8%)	1 (33.3%)
	High activity *N* (%)	10 (20.0%)	10 (50.0%)	14 (29.2%)	5 (26.3%)	1 (33.3%)
*p*		0.009 (S)		0.95 (NS)		

Fisher’s exact test was used, *S* significant, *NS* non-significant.

## Discussion

A crucial role is played by Polymorphisms in the IL17A and IL17F genes, which are genetic factors linked to the susceptibility to RA (rheumatoid arthritis), the course of the disease, and the response to treatment.^29^ The IL-17F 7488A/G polymorphism causes a His-to-Arg substitution at amino acid 161 and is found in the 3rd exon of the IL17F gene (H161R). The functional consequences of IL17F polymorphisms have been described by Kawaguchi et al.^30^ It is unlikely that the H-to-R substitution at amino acid 161 will have an impact on the protein’s core conformation. Yet, the affinity of binding to its receptor may be influenced by the substitution.^31^ This polymorphism results in a loss of the ability of IL-17F to promote the expression of specific cytokines and chemokines, which prevents the induction of IL-8 expression by wild-type IL-17F in vitro functional studies. The IL17F 7383A/G mutation results in a Glu-to-Gly substitution at amino acid 126 and may cause carriers of the uncommon G allele to have lower levels of IL-17 production and activity.^30^ Rheumatoid arthritis, autoimmune thyroid disease, prosthetic joint infection, ulcerative colitis, asthma, and malignancies have all been associated with the IL17F 7488A/G (rs763780) and IL17F 7383A/G (rs2397084) polymorphisms, according to previous research.^32–34^

Therefore, we have suggested that when IL17F expression levels are altered, genotypic variations in IL17F 7488A/G and IL17F 7383A/G polymorphisms might take part in JIA risk, hence, influencing JIA development. Owing to the disparity in the results between diverse populations, our goal was to study the association between IL17F 7488A/G and IL17F 7383A/G polymorphisms, and the risk of JIA in an Egyptian case–control study including 70 cases and 70 controls. As far as we know, this is the first investigation of IL17F 7488A/G and IL17F 7383A/G polymorphisms in JIA disease.

In our study, we have showcased that there is a significant association between the G allele of IL17F 7488A/G polymorphism and JIA susceptibility. The study has also revealed that a significantly higher risk of JIA was shown in individuals with AG genotype than in AA subjects (OR 3.02; 95% CI, 1.2–7.5; *p* = 0.017). In addition, individuals with the G allele were 2.8 folds more susceptible to JIA than the A allele (OR 2.8; 95% CI, 1.3–6.1; *p* = 0.008). Likewise, a significantly higher risk of JIA was revealed in individuals with AG genotype compared to AA subjects (OR 4.27; 95% CI, 1.59–11.4; *p* = 0.002). Moreover, individuals with the G allele were 3.72 folds more likely to have JIA than the A allele (OR 3.72; 95% CI, 1.4–9.5; *p* = 0.006).

Our results agreed with Ping et al., who conducted a meta-analysis regarding the association of Interleukin-17F 7488A/G and 7383A/G Polymorphisms with rheumatoid arthritis. In their meta-analysis, a total of seven publications with 1409 RA patients and 1303 controls were included. It was revealed in the results that IL-17F 7488A/G was significantly associated with RA, as proved by the heterozygote model (GA vs. AA), homozygote model (GG vs. AA), dominant model (GG + GA vs. AA), and recessive model (GG vs. GA + AA), which indicated that a higher risk for RA was shown with the GG and/or GA genotype holders. In addition, the 7488A/G variant might correlate with RA risk in an ethnic-specific manner, specifically in Europeans but not in Americans or Africans. Yet, regarding the relationship between IL-17F 7383A/G and RA susceptibility, no evidence was found in any genetic models either in the overall or subgroup population.^35^

IL17F 7383A/G and IL17F 7488A/G genotypic distributions and allelic frequencies of RA patients and healthy persons demonstrated statistically significant differences. These results support the findings of Marwa et al. We discovered a significant association between the risk of RA and IL17F 7383A/G. Between patients and controls, there was a substantial variation in the distribution of the IL17F 7383G allele (OR = 5.32, 95%CI = 3.22–8.84, *p* < 0.0001). IL17F 7383A/G and RA susceptibility were also observed to be significantly correlated in the dominant model (OR = 5.80, 95% CI = 3.32–10.5, *p* < 0.0001). Similarly, a strong correlation between IL17F7488 A/G and RA risk was discovered. Patients harboring at least one copy of the G allele were 6.40 times more likely to develop RA than healthy participants (OR = 6.40, 95% CI = 3.26–12.67, *p* < 0.0001); there was a significant difference in the distribution of the G allele between patients and controls (*p* = 0.00002).^36^

According to several studies, the IL17F polymorphisms His161Arg (7488 A/G) and Glu126Gly (7383A/G) are significantly correlated with the onset and progression of human illnesses. Paradowska et al. failed to discover a meaningful relationship between these two polymorphisms and RA risk in a Polish population. The differences in each population’s racial makeup, genetic heritage, and traits may account for the observed variance.^37^

Our study showed a significantly higher risk to develop high activity JIA in individuals with AG genotype of Interleukin-17F 7383A/G polymorphism compared to AA subjects (*p* = 0.009), whereas no significant association was found between genotype distributions of Interleukin-17F 7488A/G polymorphism and JIA activity (*p* = 0.95). There have been no reports in previous studies regarding the association between genotype distributions of these polymorphisms and JIA activity. To our knowledge, these polymorphisms in JIA activity have not been assessed, and there has been a focus on the primary form of RA in studies conducted on the role of Th17 and IL- 17 response. According to Marwa et al., classification based on severity revealed that individuals with the IL17F 7383A/G genotype had a greater chance of developing RA at a High Disease Activity (HDA) level compared to healthy controls (OR = 3.9, 95% CI = 1.84–8.2, *p* = 0.001). In addition, individuals with the IL17F 7488A/G genotype were found to be at an increased risk of developing RA at the HDA level compared to healthy controls (OR = 2.83, 95% CI = 1.11–6.9, *p* = 0.02).^36^ Similarly, Demircan et al., determined that there was no association between polymorphisms of the IL-17F gene and susceptibility to RA patients. Nevertheless, there was a correlation between the IL-17F variant and parameters of disease activity such as the number of tender joints and DAS-28-CRP. Furthermore, there may be a correlation between IL-17F gene polymorphism and longer disease duration in patients with RA.^38^ Yet, it was ascertained by Pawlik et al. that the factors correlated with susceptibility to RA did not include IL17A and IL17F gene polymorphisms. In addition, there were no statistically significant correlations between these polymorphisms and age of disease diagnosis, rheumatoid factor, joint erosions, or extra-articular manifestations.^39^

We have discovered in our study the association between the G allele of both IL17F 7488A/G and IL17F7383 A/G polymorphisms and the risk for JIA. We have also revealed that increased risk to develop JIA at high disease activity (HDA) level was present in individuals with AG genotype of Interleukin-17F 7383 A/G polymorphism than AA subjects. Considering the insufficient cases of JIA, future work is needed to broaden the sample size of the cases to guarantee enough statistical power for analysis. Moreover, due to our inability to measure both the synovial and serum levels of IL-17F due to financial reasons, we were unable to discover the effects of different IL-17 F gene variants s on the level of cytokine production.

## Conclusion

Children with the AG genotype of Interleukin-17F 7383A/G polymorphism had a higher risk of developing JIA at a High Disease Activity (HDA) level than AA subjects. The G allele of both IL17F 7488A/G and IL17F 7383A/G polymorphisms are associated with increased JIA susceptibility in the Egyptian population. The development of JIA appears to be affected by the polymorphisms of IL17F 7488A/G and IL17F7383 A/G. Our results underline the importance of conducting an additional study into the several gene variations associated with JIA in Egyptian children.

## Data Availability

The datasets generated during and/or analyzed during the current study are available from the corresponding author on reasonable request.
